# Redox phenotype confers T cell-exclusion microenvironment and resistance to immunotherapy by suppressing *STING*/*MDA5* expression and interferon signaling in lung cancers harboring *KEAP1*/*STK11* mutations

**DOI:** 10.3389/fonc.2025.1676797

**Published:** 2025-11-25

**Authors:** Ashish Shrestha, Yangchan Li, Lixia Huang, Shaoli Li, Yanbin Zhou, Jincui Gu, Ziying Lin

**Affiliations:** 1 Department of Respiratory and Critical Care Medicine, The First Affiliated Hospital of Sun Yat-sen University, Guangzhou, China; 2 Department of Radiation, The First Affiliated Hospital of Sun Yat-sen University, Guangzhou, China

**Keywords:** redox phenotype, KEAP1/STK11 mutations, immune microenvironment, STING, MDA5

## Abstract

**Background:**

*KEAP1* and *STK11* are frequently mutated in NSCLC, and are associated with compromised response to immunotherapy, the underlying mechanism of which is not fully understood.

**Methods:**

To assess the impact of *KEAP1*/*STK11* mutations on immune profiles, we analyzed RNA-seq data from the TCGA lung cancer cohort and the GSE72094 cohort. Differential expression, pathway enrichment, and correlation analyses were performed to elucidate the underlying mechanisms. Key findings were further validated using a single-cell RNA-seq dataset. Additionally, the prognostic significance of these mutations in immunotherapy was evaluated using immune checkpoint inhibitor (ICI) cohorts from our medical center and published studies.

**Results:**

We observed the simultaneous upregulation of pathways involved in oxidoreductase activity and down-regulation of interferon signaling pathways by mutation of *KEAP1* or *STK11*, and developed a redox signature driven by *KEAP1*/*STK11* mutations. Redox score exhibited negative correlation with expression of *STING*/*MDA5*, which function as sensors of dsDNA/dsRNA and activate downstream interferon signaling. Redox score and *STING*/*MDA5* expression manifested the exact opposite impact on the infiltrating level of most immune cells. Analysis of single cell RNA sequencing dataset indicated that redox phenotype specifically impacted expressional level of *STING*/*MDA5* in cancer cells but other cell types within tumor immune microenvironment. Prognostic significance of redox signature was validated in immunotherapy cohorts of lung cancer and melanoma, which all indicated a significant worse outcome associated with higher redox score.

**Conclusions:**

Collectively, we associated the redox status mediated by loss-function mutations of *KEAP1* or *STK11* to immune evasion and immunotherapeutic resistance by suppressing *STING*/*MDA5* expression and interferon signaling of cancer cells. Our findings link redox homeostasis to *STING/MDA5* expression and tumor immunogenicity, raising the possibility that targeting this axis could represent a future strategy to enhance ICI efficacy.

## Introduction

Non-small cell lung cancer (NSCLC), which constitutes the majority of lung cancer cases, continues to pose a significant challenge in oncology due to its aggressive nature and limited treatment options (1). The advent of immunotherapy has revolutionized cancer treatment, offering new hope for patients with advanced NSCLC (2–5). However, the response to immune checkpoint inhibitors varies widely, and understanding the underlying mechanisms of resistance is essential for improving therapeutic outcomes.

One approach to address immunotherapy resistance is to define tumor-intrinsic genetic mutations that modulate the tumor immune microenvironment (TIME) and therapy response. Among the myriad of genetic alterations that drive lung carcinogenesis, mutations in the genes like *KEAP1* (Kelch-like ECH-associated protein 1) and *STK11* (serine/threonine kinase 11) have emerged as significant players in influencing immune surveillance and therapeutic response to immune check-point inhibitors (ICIs) (6, 7). *KEAP1* is mutated in approximately 20% of lung adenocarcinomas and squamous cell carcinomas, as identified by cancer genome sequencing studies (8). It is the third most frequently mutated gene in NSCLC, encodes a protein crucial for the ubiquitination and proteasomal degradation of Nuclear factor erythroid 2-related factor 2 (NRF2), and works as a critical component during the anti-oxidant response (9–11). Its role extends beyond cellular homeostasis, as alterations in *KEAP1* function have been implicated in modulating immunogenicity and restricting the efficacy of immunotherapeutic interventions (7, 12, 13). *STK11*, also known as *LKB1* (Liver kinase B1), whose loss-function mutation occurs in approximately 10% of NSCLC, is a tumor suppressor gene that plays a critical role in cellular processes such as metabolic reprogramming, cell polarity, and proliferation (14–17). In recent studies, *STK11* loss has also been associated with reduced immune-infiltration and poor response to immunotherapy (18, 19). However, the mechanism by which *KEAP1* or *STK11* mutations impeding tumor immunogenicity and immunotherapeutic response remains elusive.

The crosstalk between *KEAP1* and *STK11* in the context of metabolic reprogramming and therapeutic resistance has been reported previously (20). For instance, loss of *LKB1* upregulates the KEAP1-NRF2 pathway, driving glutamine dependence and resistance to radiotherapy and ferroptosis (21, 22). Whether metabolic alteration is also the common mechanism shared by *KEAP1* and *STK11* in immune remodeling is yet to be evaluated. Specifically, mutations in *KEAP1* and *STK11* are both known to be involved in modulating cellular redox homeostasis. A growing body of evidence suggests that the redox phenotype of tumors, characterized by alterations in oxidative stress response pathways, can significantly influence the immunological landscape (23–25). In particular, tumors with a high redox phenotype have been associated with a T cell-exclusion microenvironment, which is generally less responsive to immunotherapeutic interventions (26). Whether alteration of redox homeostasis is the key player of immune suppression among tumors with mutant *KEAP1* or *STK11* and how it interacts with TIME is yet to be explored.

This article aims to dissect the complex relationship between *KEAP1*/*STK11* mutations, the redox homeostasis, and immune remodeling in NSCLC. Our results showed that tumors with mutant *KEAP1*/*STK11* exhibited upregulation of oxidoreductase activity and repression of interferon signaling. *KEAP1*/*STK11* mutations or redox phenotype are associated with downregulation of genes involved in dsDNA/dsRNA sensing like *STING* and *MDA5*, which leads to repression of downstream interferon signaling and immune exclusion. Redox signature was predictive of immunotherapeutic outcomes in NSCLC and other cancer type.

## Methods

### Patients’ cohort

#### Discovery cohorts

To explore the correlation between *KEAP1*/*STK11* mutations and outcomes of immunotherapy in NSCLC, we retrospectively assembled a cohort of 185 consecutive patients in our medical center who had received immunotherapy treatment and undergone molecular profiling between March 2010 and April 2023 (as detailed in **Supplementary Table 1**). Clinical data including therapeutic regiment, line of treatment, progression free survival (PFS), overall survival (OS), response to immunotherapy as accessed by Response Evaluation Criteria in Solid Tumors (RECIST) standard, mutation status of *KEAP1* and *STK11* were retrieved from medical records. Additionally, we incorporated an independent cohort from the Memorial Sloan Kettering Cancer Center and MD Anderson Cancer Center (MSKCC/MDACC cohort) (27), comprising patients with advanced NSCLC who had undergone Programmed Death- (Ligand) 1 (PD-(L)1) checkpoint blockade and comprehensive genomic profiling of their tumors (n=179). Clinical data, including treatment regimen, therapeutic outcomes, OS, PFS, and the mutation status of *KEAP1* and *STK11*, were sourced from the cBioportal database (https://www.cbioportal.org/).

In order to assess the influence of *KEAP1*/*STK11* mutations on immune profiles, we included NSCLC patients (n=1144) with available whole-exome sequencing data and genomic mutational profiles from The Cancer Genome Atlas (TCGA). Clinical information, RNA sequencing data and mutational status of *KEAP1*/*STK11* were retrieved from the cBioportal database, which can be accessed through the following link: https://www.cbioportal.org/study/summary?id=nsclc_tcga_broad_2016. Additionally, we acquired another dataset, GSE72094, comprising 441 lung adenocarcinoma tumors that had been profiled using microarray-based gene expression assays and included information on *STK11* mutations. This dataset was sourced from the Gene Expression Omnibus (GEO) under the accession number GSE72094. A comprehensive summary of the details for TCGA cohort and GSE72094 cohort were shown in **Supplementary Tables 2** and **3** respectively.

#### Single-cell RNA sequencing cohort

A published dataset (28) of single-cell RNA sequencing (scRNA-seq), which included treatment-naïve samples from 42 patients diagnosed with advanced NSCLC, was repurposed in our study as scRNA-seq cohort. Raw data of RNA sequencing, which involved 88794 single cells, was obtained from the GEO database under the accession number GSE148071. Processed data of scRNA-seq were presented in **Supplementary Table 4**.

#### Immunotherapy cohorts

In order to confirm the predictive significance of the redox signature for immunotherapy outcomes, we methodically gathered pre-treatment transcriptomic data and clinical information from two immune checkpoint inhibitor (ICI) cohorts: the Ravi lung cancer cohort (29), the Gide melanoma cohort (30). All patients within these cohorts were treated with anti-PD-1 therapy, specifically nivolumab or pembrolizumab. For the Ravi lung cancer cohort, we exclusively included cases where ICIs were administered as a first-line treatment. We collected clinical data such as PFS, OS, and the clinical response to immunotherapy, which was evaluated using the RECIST standards. A comprehensive summary of the details for all ICI cohorts is presented in **Supplementary Tables 5** and **6**.

### Assessment of immune infiltration based on RNA-seq data

Infiltrating levels of 20 leukocyte subtypes were quantified based on the expression profiles of their corresponding leukocyte signatures, which were derived from previously published research (31). The enrichment scores for each leukocyte signature were determined using the single-sample gene set enrichment analysis (ssGSEA) method, as implemented by the R-packages (GSEABase and GSVA) (32). The gene signatures for the 20 leukocyte subtypes are detailed in the **Supplementary Table 7**.

### Differential expression analysis and pathway analysis

Genes that were differentially expressed between two comparator groups—mutant *KEAP1* versus wild-type *KEAP1*, and mutant *STK11* versus wild-type *STK11*—were identified using differential expression analysis facilitated by the DESeq2 package (version 1.26.0) within the R software environment (version 3.6.3). Genes were classified as differentially expressed (DEGs) if they exhibited a log2(fold change) greater than 1 and a P-value less than 0.05. The gene lists of DEGs that were consistently up-regulated in tumors with mutant *KEAP1* and *STK11* across all datasets (TCGA-luad, TCGA-lusc, GSE72094) were subjected to Gene Ontology (GO) analysis. This analysis was conducted using the ‘msigdbr’ package (version 7.5.1) and the clusterProfiler package (version 4.12.6) within the R software environment (version 4.4.1). Conversely, DEGs that were down-regulated in both mutant *KEAP1* and *STK11* tumors were analyzed using Reactome pathway analysis, which was implemented with the ReactomePA R package (version 1.48.0).

### Development of redox signature and scoring pipeline

Differential expression analysis was performed to identify genes upregulated by mutant *KEAP1* in the TCGA-LUAD and TCGA-LUSC cohorts, as well as those upregulated by mutant *STK11* in the TCGA-LUAD and GSE72094 cohorts. From these analyses, we identified 148 overlapping genes common to all four upregulated gene sets. These genes were subsequently subjected to GO pathway enrichment analysis, which revealed that the top 8 enriched pathways were associated with oxidoreductase activity and antioxidant functions. Further refinement of the 148-gene pool identified 26 genes (**Supplementary Table 7**) implicated in these 8 pathways, which we designated as the redox signature. To quantify the enrichment of this redox signature in individual tumors, we applied the single-sample Gene Set Enrichment Analysis (ssGSEA) algorithm. This method ranks genes based on expression and calculates a normalized enrichment score (NES) for each sample, eliminating the need for batch adjustment. The ssGSEA analysis was performed using the normalized gene expression matrix and an unweighted redox gene list as input, implemented via the R packages GSEABase and GSVA (see Supplementary Material for R code).

### Single-cell sequencing analysis

Pre-processed RNA sequencing data of single cells derived from 42 treatment naïve NSCLC tumors were obtained from the public available dataset GSE148071. The Seurat package (version 4.4.1) was employed to create the object, filtering out cells of poor quality based on the following criteria: lower than 200 or higher than 5000 expressed genes, and cells with >20% mitochondrial content. Expression matrices were normalized by function NormalizeData and ScaleData. The top 2000 variable genes, identified by the FindVariableFeatures function, were used for principal components analysis. The first 30 principal components and resolution 0.6 were used with FindClusters function to generate 30 cell clusters, which was assigned to 8 major cell types based on the expression pattern of the following canonical markers: Endothelial cells (PECAM1, FLT1, VWF), cancer cells (EPCAM, KRT19, SOX2, EGFR), Alveolar cells (CLDN18, AQP4, SFTPC), Fibroblasts (COL1A1, COL1A2, DCN), T cells (CD2, CD3D, TRAC, NKG7), B cells (CD79A, IGHG3, IGHA2), Myeloid cells (CD68, CD14, LYZ), Neutrophils (CSF3R, FCGR3B). All these cluster markers were decided based on reference to previous publication (28, 33) and CellMarker dataset (http://bio-bigdata.hrbmu.edu.cn/CellMarker/). Bulk expression profiles of each tumor were inferred based on the average expression profiles of all the cells derived from the same tumor. AverageExpression function was used to generated averaged expression profiles of each cellular subset.

### Statistical analysis

**Figure 1:**
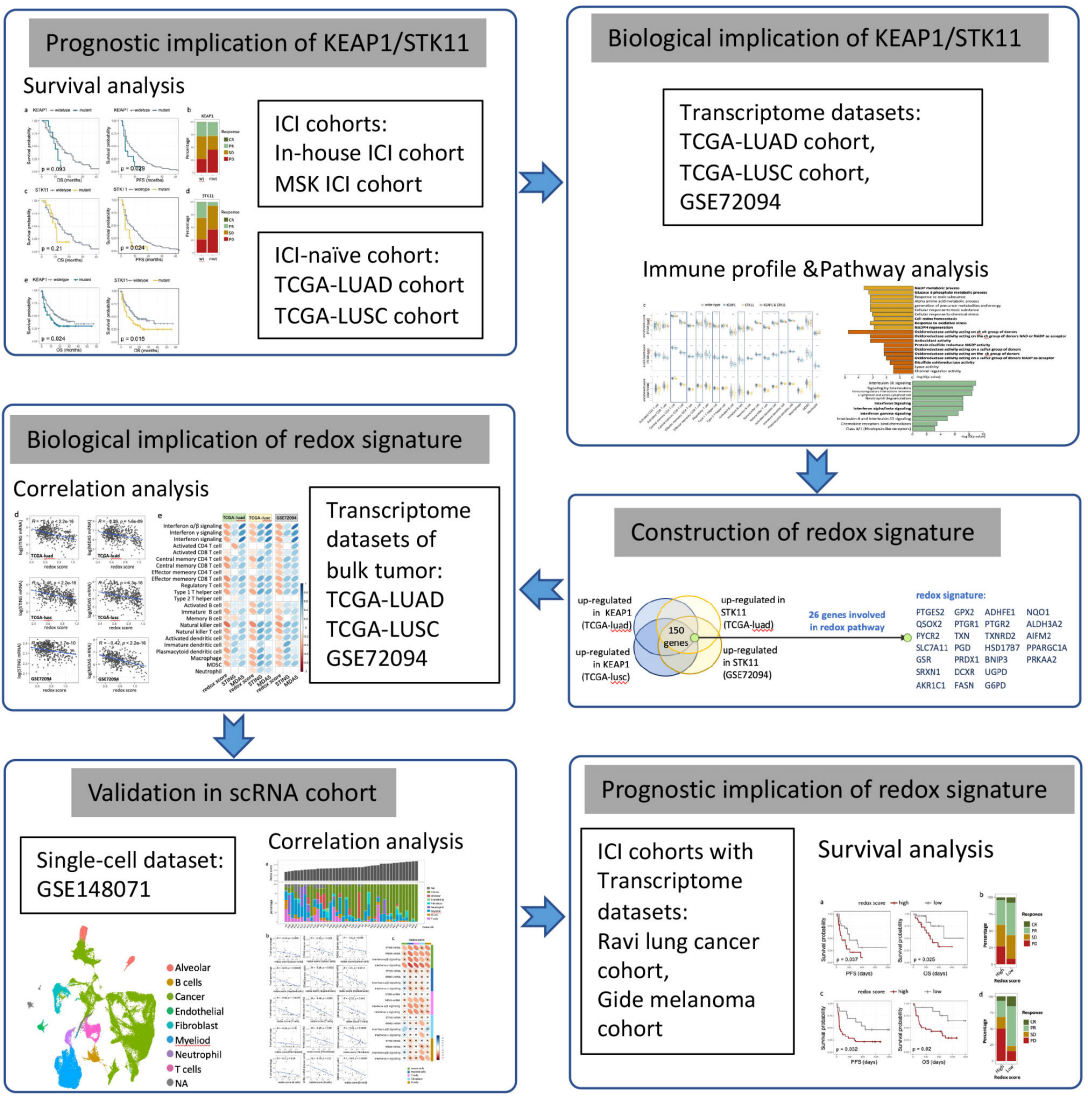
Flow diagram demonstrating the outlook of the study.

Statistical analyses were performed using R v4.4.1 (https://www.r-project.org) or SPSS software (v26). Comparison of enrichment scores or gene expression level between two groups was analyzed by two-sided Wilcoxon tests. Spearman correlation analysis was applied to evaluate correlation between two continuous parameters. Distribution of categorical data between two groups were analyzed using the chi-square test. Kaplan-Meier curve was used to estimate median OS and PFS, with statistical difference between two groups accessed by log-rank test. Univariate and multivariate Cox regression analyses were performed to assess the prognostic significance of multiple variables. Similarly, univariate and multivariate logistic regression analyses were conducted to evaluate the influence of different variables on the clinical response to immune checkpoint inhibitors (ICIs). A two-sided p-value of less than 0.05 was considered statistically significant. The study design and key statistical analyses were summarized in a flow diagram, as illustrated in **Figure 1**.

## Results

### *KEAP1*/*STK11* mutations diminishes immunotherapy efficacy in NSCLC

**Figure 2:**
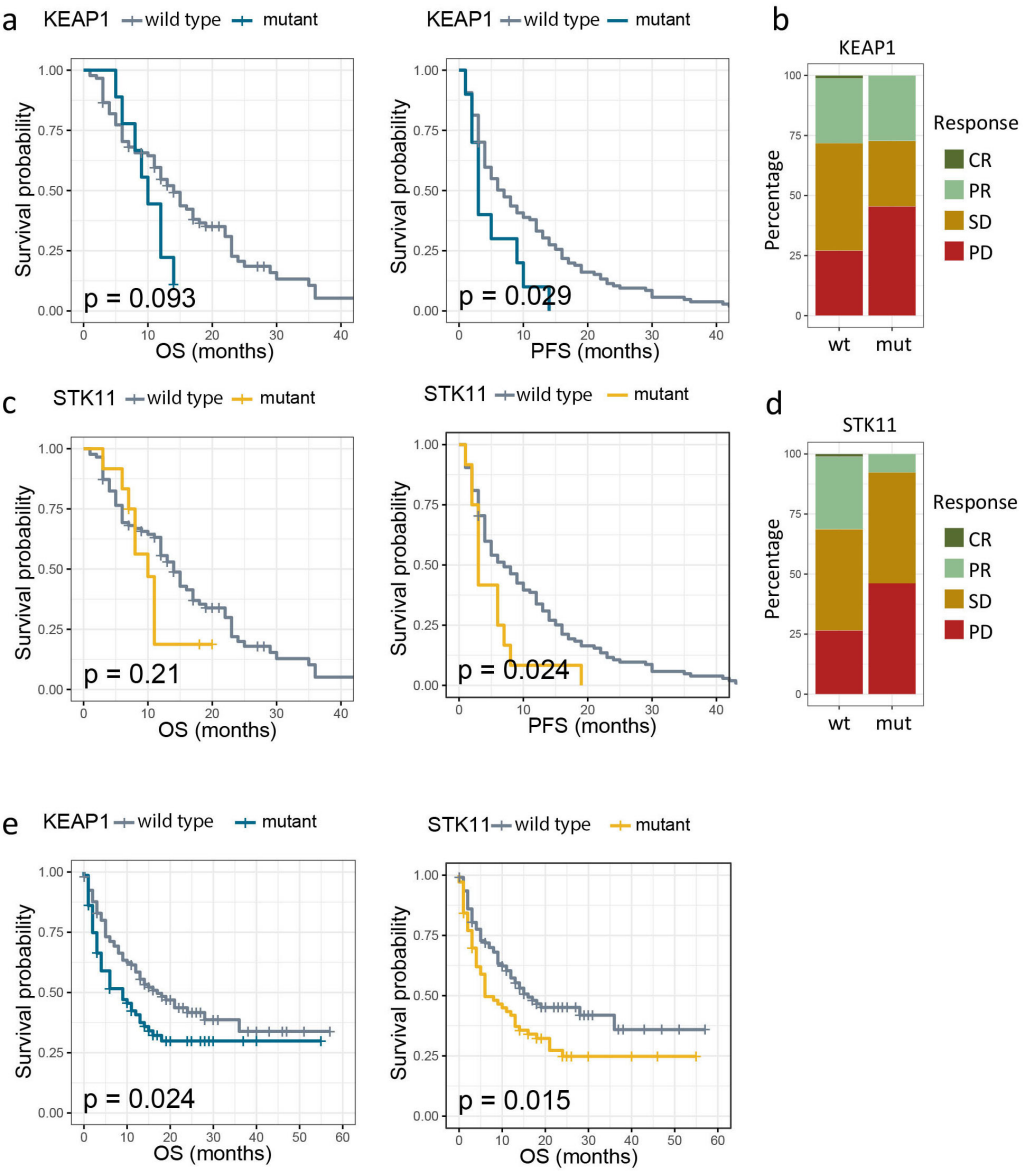
Association between mutation status of *KEAP1*/*STK11* and therapeutic outcomes to immunotherapy. **(a, c).** Kaplan–Meier survival curves of progression-free survival (PFS) and overall survival (OS) between patients with wild-type and mutant *KEAP1***(a)**, as well as between patients with wild-type and mutant *STK11***(c)** in our own immunotherapy cohort; **(b, d)** Bar charts showing the distribution of patients with complete response (CR), partial response (PR), stable disease (SD) or progression disease (PD) between patients with wild-type and mutant *KEAP1* (a), as well as between patients with wild-type and mutant *STK11***(c)** in our own immunotherapy cohort; **(e)** Kaplan–Meier survival curves of OS by mutational status of *KEAP1* or *STK11* among NSCLC patients from MSK immunotherapy cohort.

Mutations in *KEAP1* and *STK11* had been frequently reported to diminish immunotherapy efficacy (6, 7). An in-house cohort comprising 185 patients of advanced NSCLC treated with PD-1/PD-L1 immunotherapy alone or in combination with chemotherapy was deployed to verify the prognostic impact of mutant *KEAP1*/*STK11*. In line with previous studies (6, 7), we found that patients harboring mutant *KEAP1* or *STK11* had significant inferior PFS as compared to those with wild-type *KEAP1*/*STK11* (**Figures 2a, c**). The proportion of patients achieving complete response (CR) or partial response (PR) was markedly lower in patients with mutant *KEAP1*/*STK11* as compared to those with wild-type variants (**Figures 2b, d**). Although not statistically significant in our cohort, patients with *KEAP1*/*STK11* mutations tended to have reduced OS (**Figures 2a, c**). Analysis of an additional immunotherapy cohort, the MSK lung cancer cohort, corroborated our findings, demonstrating that mutations in *KEAP1* or *STK11* were linked to unfavorable outcomes, as evidenced by significantly poorer OS (**Figure 2e**). It is noteworthy that *KEAP1* and *STK11* did not affect survival in NSCLC patients who underwent surgical treatment (**Supplementary Figures 1a, b**), suggesting that their prognostic significance is specific to the context of immunotherapy.

### NSCLC with mutant *KEAP1* or *STK11* exhibited immunosuppressive microenvironment in a similar pattern

**Figure 3:**
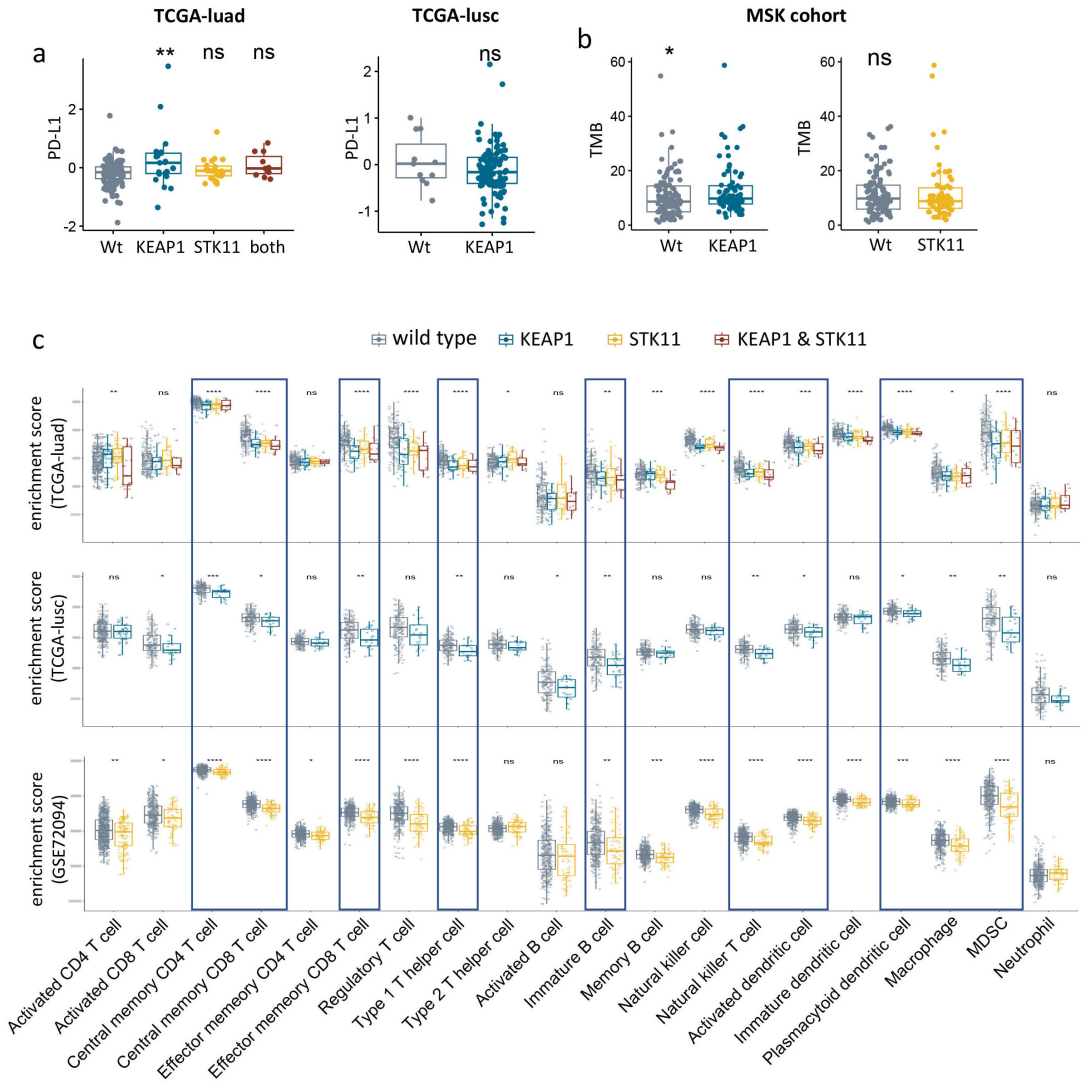
Immunologic consequences of *KEAP1*/*STK11* mutations. **(a)** Boxplots showing PD-L1 protein expression level in NSCLC with mutant or wild-type *KEAP1*/*STK11*. Analysis was performed in two cohorts (TCGA-luad, TCGA-lusc). **(b)** Boxplots showing TMB level of *KEAP1*/*STK11* mutant or wild-type tumors from MSK immunotherapy cohort. **(c)** Boxplots showing infiltrating abundance of 20 immune cells among tumors with mutant or wild-type *KEAP1*/*STK11*. Analysis was performed in three cohorts (TCGA-luad, TCGA-lusc, GSE72094). **(a, c)** As for TCGA-luad cohort, tumors were categorized into four groups based on mutational status of *KEAP1* and *STK11*. Analysis only involved mutational status of *KEAP1* or *STK11* in TCGA-lusc cohort and GSE72094 cohort respectively, owing to insufficiency or unavailability of relevant data. **(a–c)** Data are presented as median with quartiles. Wilcoxon tests was used to determined significance in difference between two groups. Kruskal -Wallis was performed for multiple comparison. *P < 0.05, **P < 0.01, ***P < 0.001, ****P < 0.0001.

We subsequently investigated the influence of *KEAP1*/*STK11* mutations on established biomarkers of immunotherapeutic response, such as PD-L1 expression and tumor mutation burden (TMB). In our analysis of the TCGA lung cancer dataset, where protein expression levels were accessible, we found no significant differences in PD-L1 expression levels between tumors harboring wild-type and mutant *KEAP1*/*STK11*, both in adenocarcinoma (TCGA-LUAD) and squamous cell carcinoma (TCGA-LUSC) (**Figure 3a**). Furthermore, TMB was not affected by *STK11* mutations; and in fact, it was even elevated in tumors with mutant *KEAP1* according to our analysis of the MSK lung cancer dataset (**Figure 3b**). These observations suggest that the poor response to immunotherapy in patients with mutant *KEAP1*/*STK11* is not due to reduced PD-L1 expression or TMB levels, but rather may be attributed to alterations in the immune microenvironment.

To access the impact of *KEAP1*/*STK11* mutations on immune landscape of lung cancer, we utilized two bulk RNA-seq datasets: TCGA-lung cancer and GSE72094, for our analysis. The infiltration levels of 20 different leukocyte populations were deduced from the enrichment scores of their respective gene signatures (as detailed in **Supplementary Table 7**). **Figure 3c** illustrates that lung tumors with mutations in *KEAP1* or *STK11* displayed an immunosuppressive phenotype, characterized by a significant reduction in the infiltration of a broad spectrum of leukocytes. These included central memory CD4 T cells, central memory CD8 T cells, effector memory CD8 T cells, type 2 T helper cells, immature B cells, natural killer T cells, activated dendritic cells, plasmacytoid dendritic cells, macrophages, and myeloid-derived suppressor cells (MDSCs). Due to the limited number of tumors with detecs. Table *TK11* mutations (only three), the analysis of *STK11* status in the TCGA-LUSC cohort was not conducted. Similarly, the assessment of *KEAP1* status in the GSE72094 cohort was not possible due to the absence of pertinent data. It’s intriguing to notice that the impact of mutant *KEAP1* and *STK11* on the infiltration levels of various leukocytes was similar, which indicated *KEAP1* and *STK11*might employ analogous mechanisms in immune regulation.

### *KEAP1*/*STK11* mutations confer redox phenotype and suppression of IFN signaling in lung cancer

**Figure 4:**
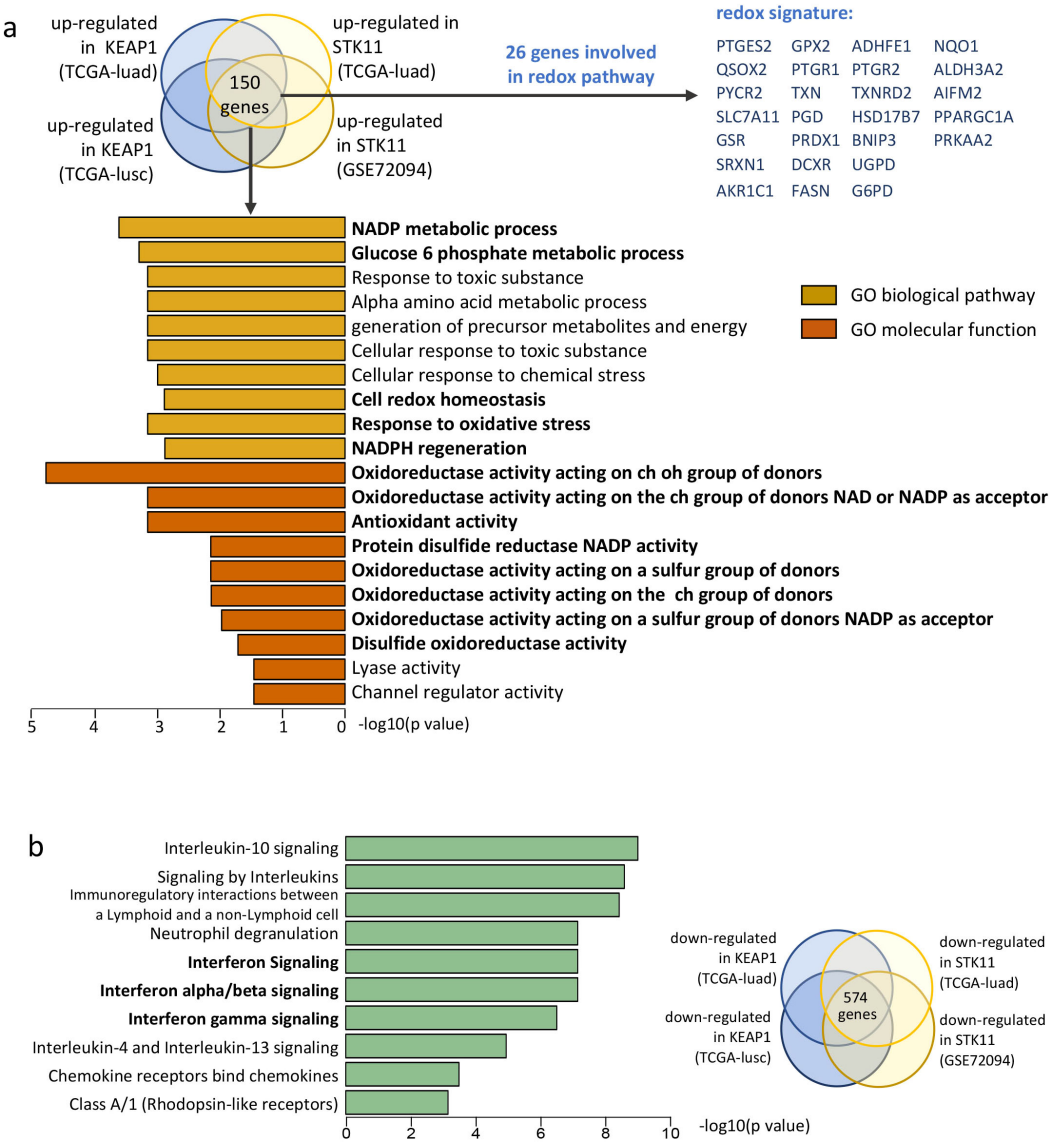
Pathway analysis reveals enrichment of redox pathways and down-regulation of IFN signaling in NSCLC with *KEAP1*/*STK11* mutations. **(a)** Gene signature enrichment analysis (GSEA) was performed for the overlapped up-regulated genes based on Gene Ontology (GO) categories. Diagram on the top showing the generation of overlapped genes up-regulated by *KEAP1* and *STK11* among different cohorts. A total 150 overlapped genes identified, among which 26 genes were identified to be involved in redox biological process. Bar plot showing the top 10 most significantly enriched pathways for the GO-biological pathway category and GO-molecular function category respectively. **(b)** Gene signature enrichment analysis (GSEA) was performed for the overlapped down-regulated based on Reactome categories. Diagram on the right showing the generation of overlapped genes down-regulated by *KEAP1* and *STK11* among different cohorts, with a total 574 overlapped genes identified. Bar plot showing the top 10 most significantly enriched pathways.

To investigate the shared biological processes influenced by *KEAP1* and *STK11* mutations, we focused on the 151 differentially expressed genes that were consistently up-regulated by these mutations across all cohorts. These genes were subjected to Gene Ontology (GO) pathway analysis. **Figure 3a** displays the top 10 GO biological pathways (GOBP) and top 10 GO molecular functions (GOMF) that were concurrently up-regulated by mutant *KEAP1* and *STK11*. Notably, the majority of the up-regulated pathways were associated with oxidoreductase activity, suggesting that a redox phenotype is the predominant feature in tumors with mutant *KEAP1* or *STK11*. Among the 148 overlapping up-regulated genes, 25 were identified as being involved in redox activity and were selected to form a redox signature for further investigation (**Figure 4a**). Spearman correlation analysis revealed that all 26 genes within the redox signature were significantly and negatively correlated with the infiltration levels of most leukocytes, particularly those that were markedly reduced in tumors with mutant *KEAP1* or *STK11* (**Supplementary Figure 2a**). This correlation suggests that the redox phenotype may be a critical factor driving immune evasion in tumors harboring *KEAP1* or *STK11* mutations.

We calculated the enrichment score of the redox signature for each tumor using the ssGSEA method. As depicted in **Supplementary Figure 2b**, the redox score did not demonstrate prognostic relevance among NSCLC patients who underwent surgical treatment. Specifically, there was no observed difference in OS or PFS between patients with high and low redox scores within the TCGA cohorts (TCGA-LUAD and TCGA-LUSC). We proceeded to assess the correlation between the redox score and various clinicopathological characteristics, including histology, gender, smoking history, and TNM stage. The redox score was significantly elevated in tumors originating from squamous cell carcinoma as compared to adenocarcinoma, and also in tumors from male patients as compared to female patients (**Supplementary Figure 2c**). However, neither smoking history nor TNM staging had a significant influence on the redox status of NSCLC tumors (**Supplementary Figure 2c**).

To elucidate the mechanism through which the redox phenotype facilitates immune evasion in NSCLC with mutations in *KEAP1* or *STK11*, we conducted a further analysis to identify pathways enriched in genes that are down-regulated by these mutations. We identified a total of 574 down-regulated genes that were common across all cohorts. These genes were then subjected to Reactome pathway enrichment analysis. Notably, three of the top 10 enriched pathways were associated with the activation of interferon signaling, including the pathways for interferon α/β signaling and interferon γ signaling (**Figure 4b**).

### Redox phenotype mediates immune exclusion by repressing *STING*/*MDA5* expression and interferon signaling

**Figure 5:**
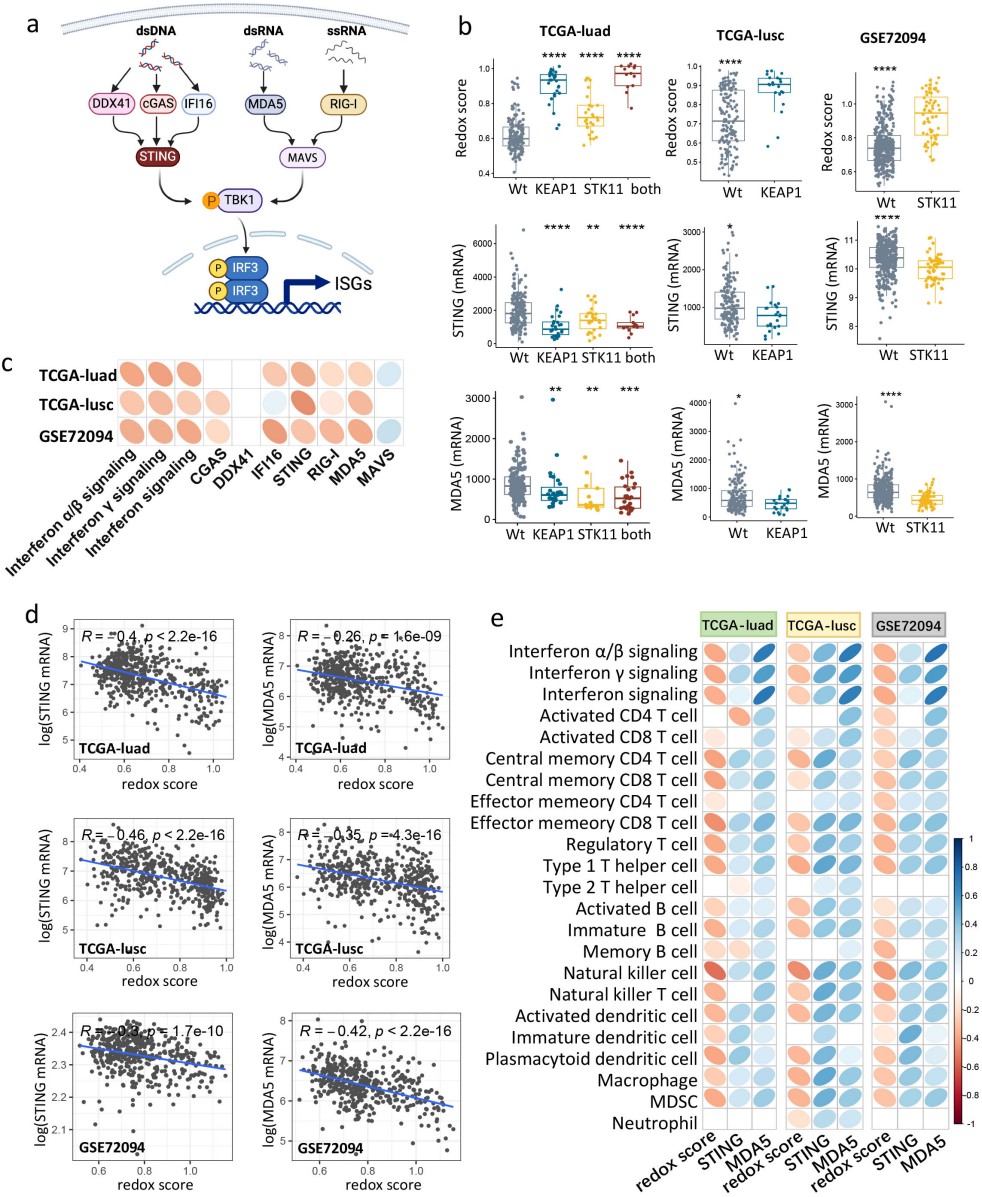
Redox scoring negatively correlated with *STING*/*MDA5* expression, IFN signaling and immune infiltration in NSCLC. **(a)** Schematic of dsDNA, dsRNA, ssRNA sensing pathways that induce IFN signaling. **(b)** Boxplots showing *STING*/*MDA5* mRNA expression and redox score among tumors with mutant or wild-type *KEAP1*/*STK11* in three cohorts (TCGA-luad, TCGA-lusc, GSE72094). As for TCGA-luad cohort, tumors were categorized into four groups based on mutational status of *KEAP1* and *STK11*. Analysis only involved mutational status of *KEAP1* or *STK11* in TCGA-lusc cohort and GSE72094 cohort respectively, owing to insufficiency or unavailability of relevant data. Data are presented as median with quartiles, and Wilcoxon tests was used to determined significance in difference between wild-type group and other groups. *P < 0.05, **P < 0.01, ***P < 0.001, ****P < 0.0001. **(c)** Correlation matrix depicting correlation of redox score with IFN signaling and genes involved in dsDNA/dsRNA/ssRNA sensing in three datasets (TCGA-luad, TCGA-lusc and GSE72094). **(d)** Scatter plot showing the correlation between redox score and mRNA expression of *STING*/*MDA5* in three datasets (TCGA-luad, TCGA-lusc and GSE72094). Correlation coefficients (r value) and P value of Spearman Correlation were shown. **(e)** Correlation matrix depicting correlation of redox score and mRNA expression of *STING* with infiltrating level of 20 immune cells in three datasets (TCGA-luad, TCGA-lusc and GSE72094). **(c, e)** Spearman Correlation analysis was performed, with blue ellipse obliquely upward representing positive correlation, and red ellipse obliquely downward representing negative correlation. The flatness of ellipse and the depth of the color represent the magnitude of the correlation (r value). Ellipse was presented only for those with significant correlation (P value < 0.05).

Cell-autonomous interferon responses are typically regulated by pathways involving in sensing double-stranded DNA (dsDNA), double-stranded RNA (dsRNA), or single-stranded RNA (ssRNA) (**Figure 5a**) (34, 35). As redox imbalance had been associated with DNA damage (36), we first evaluated signaling of DNA repair in tumors with *KEAP1*/*STK11* mutations. Of note, biological pathways of DNA repair or double-strand break repair were unaltered by mutations in either *KEAP1* or *STK11* (**Supplementary Figure 3**). We then proceeded to examine the differential expression of genes along the signaling axes of dsDNA/dsRNA/ssRNA sensing in tumors with wild-type versus mutant *KEAP1*/*STK11*. **Figure 5b** and **Supplementary Figures 4a–c** reveal that mRNA expression levels of *STING* (Stimulator of Interferon Genes), *MDA5* (Melanoma Differentiation-Associated protein 5) and *RIG-I* (Retinoic acid-inducible gene I) are significantly downregulated in tumors with mutant *KEAP1* or *STK11* across all cohorts. We also noted a reduction in *CGAS* (Cyclic guanosine monophosphate-adenosine monophosphate synthase) expression due to *KEAP1*/*STK11* mutations in the TCGA-LUSC and GSE72094 cohorts, but not in the TCGA-LUAD cohort. Similarly, *IFI16* (Interferon Gamma Inducible Protein 16) expression was downregulated by *KEAP1*/*STK11* mutations in the TCGA-LUAD and GSE72094 cohorts, yet this effect was not observed in the TCGA-LUSC cohort.

We next determined the correlation between the redox phenotype and IFN signaling, as well as genes involved in dsDNA/dsRNA/ssRNA sensing. As illustrated in **Figure 5c**, the redox score was found to be negatively correlated with the enrichment scores of all interferon signaling pathways. Notably, the expression levels of genes involved in dsDNA/dsRNA/ssRNA sensing, specifically *STING*, *MDA5* and *RIG-I*, were negatively correlated with the redox score across all datasets (**Figures 5c–d**). *CGAS* and *IFI16* also showed a negative correlation with the redox score, but this was only observed in certain cohorts (**Figure 5c**).

We then assessed the correlation between genes involved in dsDNA/dsRNA/ssRNA sensing and the immune profiles of NSCLC tumors using Spearman correlation analysis. As shown in **Supplementary Figure 5d**, mRNA expression of dsDNA sensors (*STING*, *CGAS*) or dsRNA/ssRNA sensors (*MDA5*, *RIG-I*) were significantly and positively correlated with the infiltrating abundance of the majority of immune cells across all cohorts. The positive correlation between *IFI16* expression and immune infiltration was observed exclusively in the adenocarcinoma cohorts (GSE72094 and TCGA-LUAD) (**Supplementary Figure 5d**). Collectively, these findings suggest that *KEAP1*/*STK11* mutations are associated with the downregulation of genes involved in dsDNA/RNA sensing, particularly *STING* and *MDA5*, which may be a key driver of immune evasion.

Given that *STING* and *MDA5* are the downstream components in dsDNA/dsRNA sensing and trigger the activation of IFN signaling, their suppression could be a pivotal mechanism by which the redox phenotype drives immune evasion in lung cancer. Specifically, we observed an inverse relationship between *KEAP1*/*STK11* mutations and changes in redox scores and *STING*/*MDA5* expression levels across all cohorts (**Figure 5b**). Correlation analysis further indicated that the redox score and *STING*/*MDA5* expression had opposite effects on the infiltration levels of nearly all immune cells across all cohorts (**Figure 5e**). It is noteworthy that the immune cells that were significantly reduced in tumors with *KEAP1*/*STK11* mutations were also the ones that showed significant positive and negative correlations with *STING*/*MDA5* expression and redox scores, respectively (**Figure 5e**). Collectively, our findings suggested that the redox phenotype, driven by *KEAP1*/*STK11* mutations, promotes immune evasion by downregulating genes involved in dsDNA/dsRNA sensing especially *STING* and *MDA5*, and thus suppresses the downstream interferon signaling pathway.

### scRNA-seq analysis reveals redox phenotype specifically impacts *STING*/*MDA5* expression of cancer cells

To identify the predominant cellular subtypes contributing to the redox status, we utilized single-cell RNA sequencing (scRNA-seq) data from 42 NSCLC tumors for further analysis. Cell clusters were classified into T cells, B cells, myeloid cells, neutrophils, fibroblasts, endothelial cells, alveolar cells, and cancer cells based on the expression of canonical marker genes (**Supplementary Figures 5a–b**). Notably, most redox-associated genes were found to be highly expressed across various cell types (**Supplementary Figure 4c**), although a slightly higher redox score was observed in cancer cells compared to other cell types (**Supplementary Figure 5d**).

**Figure 6:**
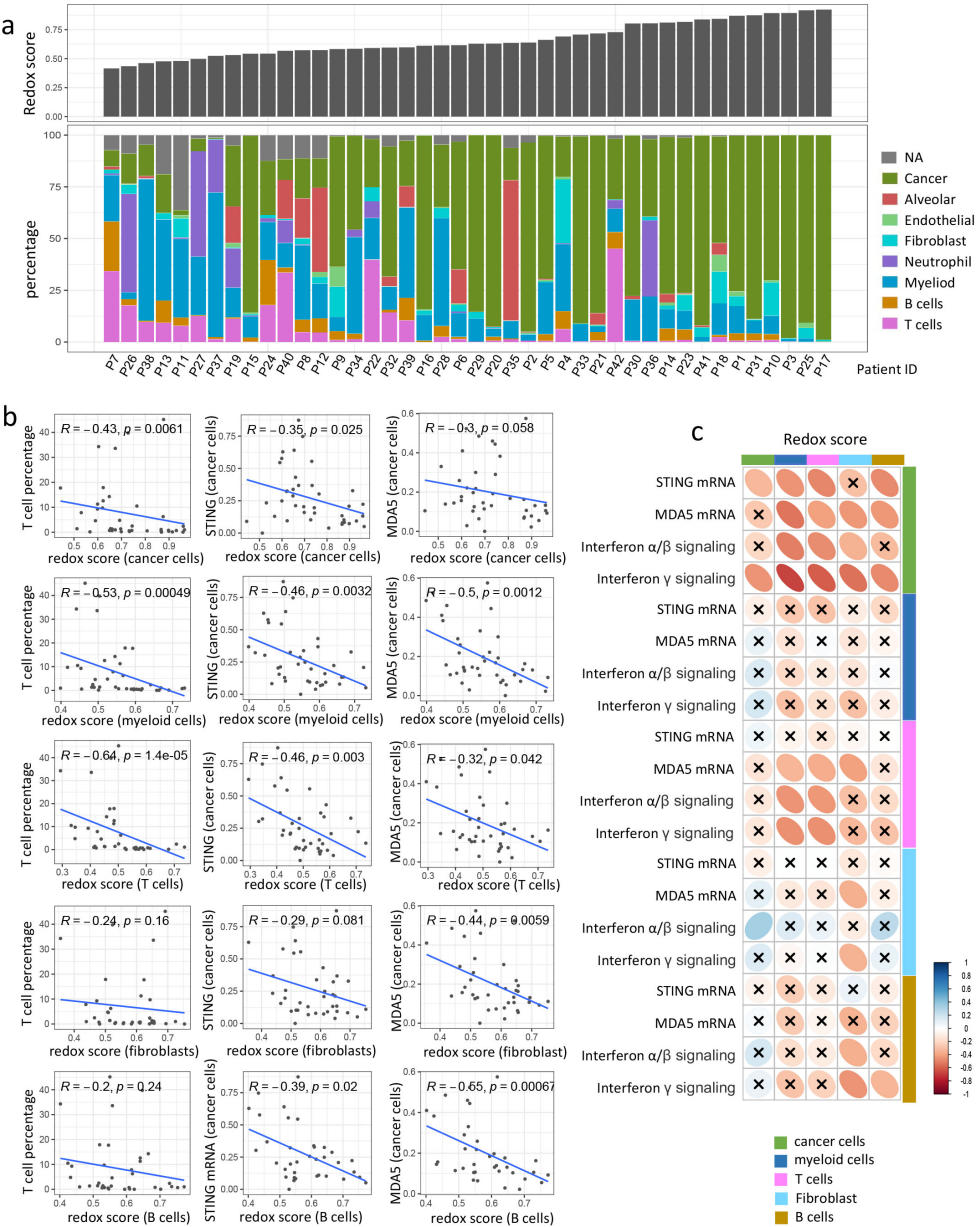
scRNA analysis reveals redox phenotype down-regulates *STING*/*MDA5* expression in cancer cells and facilitates immune exclusion. **(a)** Barplots showing bulk redox score and proportion of different cellular components in each tumor. Tumors were arranged according to bulk redox score in ascending order. **(b)** Scatter plot showing the correlation of T cell percentage or *STING*/*MDA5* mRNA expression in cancer cells with redox score of different cellular components. Correlation coefficients (r value) and P value of Spearman Correlation were shown. **(c)** Correlation matrix showing correlation of redox score with *STING*/*MDA5* mRNA expression or IFN signaling (Interferon α/β signaling and Interferon γ signaling) across different cell types. Spearman Correlation analysis was performed, with blue ellipse obliquely upward representing positive correlation, and red ellipse obliquely downward representing negative correlation. The flatness of ellipse and the depth of the color represent the magnitude of the correlation (r value). The cross mark represents the failure of the significance test (P value > 0.05).

We then assessed the impact of the redox phenotype on the immune composition within the TIME. As depicted in **Figure 6a**, the percentage of various immune cells decreased with an increase in the redox score of bulk tumors. To determine redox status of which cellular subtype plays a major role in shaping the immunosuppressive microenvironment, we calculated the redox score for five major cell types (cancer cells, myeloid cells, T cells, fibroblasts, and B cells) based on the averaged expression profiles for each tumor. **Figure 5b** shows that the redox scores of both myeloid cells and T cells were significantly negatively correlated with the T cell percentage. The redox scores of cancer cells, fibroblasts, and B cells also exhibited a negative, albeit not statistically significant correlation with the T cell percentage.

We further explored the changes in expressional level of genes involved in dsDNA/dsRNA/ssRNA sensing and the enrichment of downstream interferon signaling pathway across different cell types in response to the redox status of various cell types. It is intriguing to observe that *STING*/*MDA5* expression in cancer cells showed a negative correlation with the redox scores of nearly all major cellular subtypes, including cancer cells, myeloid cells, T cells, fibroblasts, and B cells (**Figures 6b–c**). *MDA5* expression in T cells were also negatively correlated with redox score of myeloid cells, T cells and fibroblast. However, the *STING*/*MDA5* expression of other cell types exhibited no significant correlation with redox score of any cell types (**Figure 6c**). As for other genes involved in dsDNA/dsRNA/ssRNA sensing like *CGAS, RIG-I, MAVS, DDX41, and IFI16*, whose expression in either cancer cells or other cell types, were not significantly impacted by redox status of any cell types (**Supplementary Figure 5e**). Additionally, interferon α/β signaling and interferon γ signaling in cancer cells were negatively influenced by the redox status of nearly all cellular subtypes (**Figure 6c**). Interferon signaling in T cells also negatively correlated with the redox scores of T cells and myeloid cells (**Figure 6c**). Nevertheless, no significant correlation was observed between interferon signaling and redox scores in most other cell types (**Figure 6c**). Collectively, these findings suggest that the redox phenotype mediates immune exclusion primarily by suppressing *STING*/*MDA5* expression and interferon signaling in cancer cells.

### Redox signature predicts response to immunotherapy

**Figure 7:**
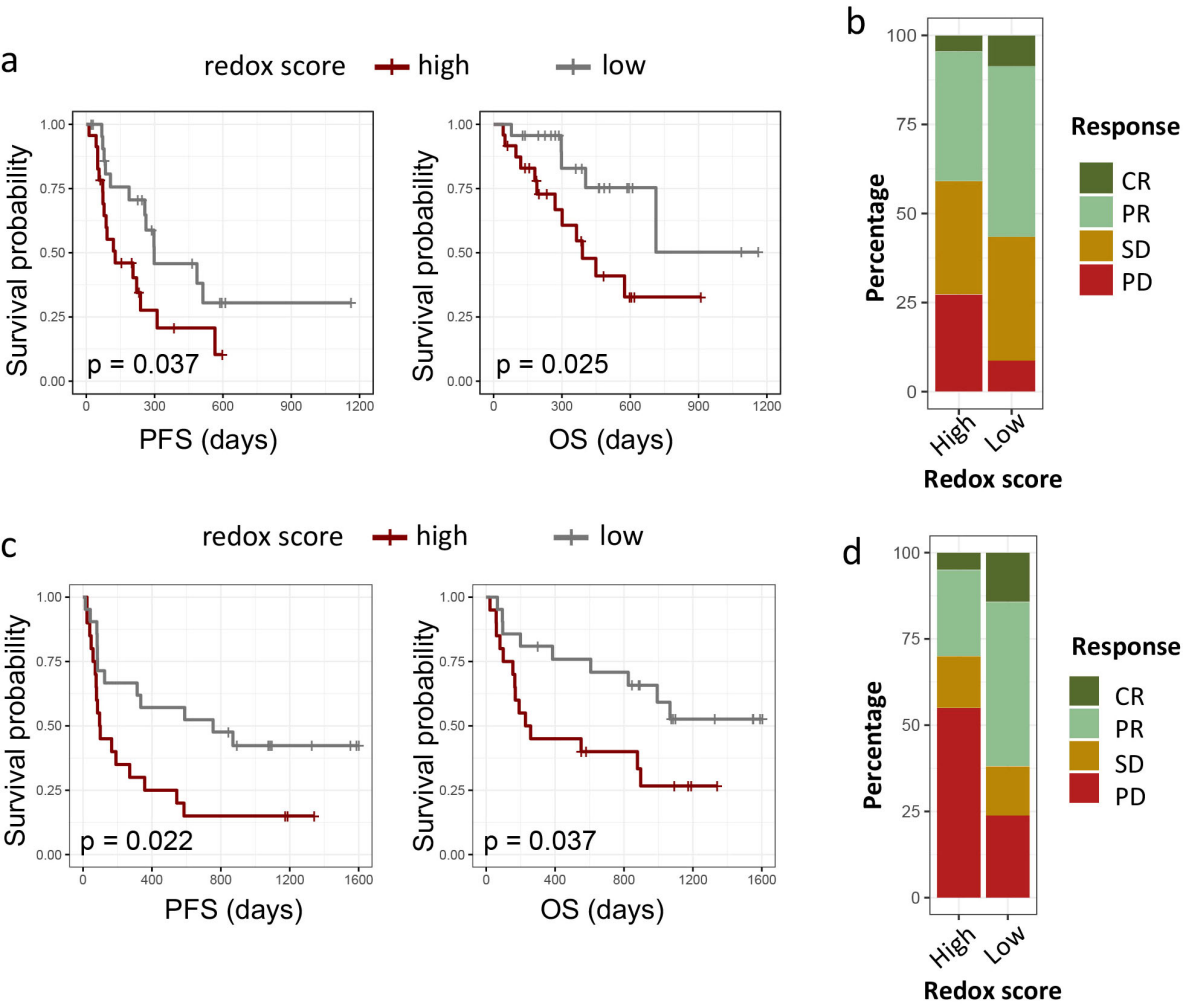
Impact of redox phenotypes on response to ICIs in multiple cancers. **(a, c)**. Kaplan–Meier survival curves of PFS and OS between high redox group and low redox group in Ravi lung cancer cohort **(a)** and Gide melanoma cohort **(c)**. **(b, d)** 100-percent bar plots showing the distribution of patients with complete response (CR), partial response (PR) stable disease (SD) or progression disease (PD) between high redox group and low redox group in Ravi lung cancer cohort **(b)** and Gide melanoma cohort **(d)**. Tumors were categorized into high and low redox group with medium redox score as cut-off value for both cohorts.

To ascertain the prognostic relevance of redox signatures in the context of immunotherapy, we assessed the relationship between redox scores and the efficacy of PD-1/PD-L1 checkpoint inhibitors in two distinct patient cohorts: the Ravi lung cancer cohort, the Gide melanoma cohort. In the Ravi lung cancer cohort, we enrolled 48 patients with advanced NSCLC who were treated with PD-L1 blockade as their first-line therapy. As depicted in **Figure 7a**, patients with lower redox scores exhibited significantly extended PFS and OS compared to those with higher redox scores. Specifically, the median PFS was 529.4 days for the low redox group versus 221.8 days for the high redox group (p = 0.037), and the median OS was 843.7 days for the low redox group versus 491.2 days for the high redox group (p = 0.025). Cox regression analysis, adjusting for PD-L1 expression (protein level), smoking status, gender, age, histology (adenocarcinoma vs. squamous), and co-mutations (KRAS, TP53, KEAP1, STK11), further confirmed redox status as the only significant predictor of reduced OS (HR 3.60 [1.54 -8.41], P = 0.00306) and PFS (HR 3.12 [1.09-8.94], P = 0.0337) in univariate analysis but not multivariate analysis (**Supplementary Tables 8.1**, **8.2**). In terms of response rates, 55% of patients with low redox scores achieved a complete response (CR) or partial response (PR), contrasted with 37% in the high redox group (**Figure 7b**). However, logistic regression analysis indicated that none of the above-mentioned variables, including redox status, were significant predictors of clinical response (**Supplementary Table 8.3**).

To evaluate the broader applicability of redox signatures in predicting responses to immunotherapy, we examined the Gide melanoma cohort. The Gide melanoma cohort comprised 41 melanoma patients who underwent anti-PD-1 treatment. Similarly, we observed a marked improvement in PFS and OS for patients with lower redox scores: the median PFS was 967 days for the low redox group versus 402 days for the high redox group (p = 0.04), and the median OS was 1278 days for the low redox group versus 616 days for the high redox group (p = 0.038) (**Figure 7c**). Cox regression analysis was limited to redox level, PD-L1 expression (mRNA level), smoking status, gender, and age, as data on other parameters were unavailable. As shown in **Supplementary Table 9**, both high redox level (HR for PFS 3.60 [1.54 -8.41], P = 0.00306; HR for OS 2.68 [1.08-6.63], P = 0.0328) and low PD-L1 expression (HR for PFS 0.14 [0.06 -0.33], P < 0.001; HR for OS 0.16 [0.06-0.46], P < 0.001) were significant independent predictor of reduced OS and PFS. Additionally, patients with lower redox scores demonstrated a higher objective response rate (ORR), with 84% achieving CR/PR in the low redox group, compared to 32% in the high redox group (**Figure 7d**). Multivariate logistic regression analysis also confirmed redox level (OR 0.16 [0.02-0.8], P = 0.04) and low PD-L1 expression (OR 15.79 [3.10-130.96], P = 0.0027) as independent predictor of clinical response to immunotherapy (**Supplementary Table 9.3**).

## Discussion

With this study, we demonstrated that NSCLC with *KEAP1* or *STK11* mutation manifested enhanced redox phenotype and diminished immune infiltration. Redox status is associated with inhibition of interferon signaling, which could be attributed to downregulation of genes involved in dsDNA/dsRNA sensing like *STING* and *MDA5* in cancer cells. Redox score and *STING*/*MDA5* expression exhibited the exact opposite correlation with infiltrating level of different immune cells. Our study suggested that *KEAP1* and *STK11* shared common mechanism in immune regulation, which is associated with enhancement of redox phenotype and the subsequent inhibition of *STING*/*MDA5* expression and the downstream interferon signaling in cancer cells. We also developed a redox signature which may be helpful in predicting outcomes to ICI treatment in NSCLC and other cancers.

Repression of type I interferon signaling in tumors harboring *KEAP1* or *STK11* mutations has been reported in previous studies (37, 38), yet the underlying mechanism is not well characterized. Cell-autonomous type I interferon responses are typically regulated by dsDNA/dsRNA sensing pathways, whose activation can be driven by overproduction of dsDNA or dsRNA, or overexpression of genes along these signaling axes (34, 35). Previous study reported that *KEAP1* mutation resulted in upregulation of *BRCA1*, which is an important DNA damage repair gene, and thus reduced production of dsDNA (39). Yet our finding suggested neither *KEAP1* nor *STK11* interfered with DNA repairing in NSCLC. Instead, several genes involved in dsDNA/dsRNA sensing like *STING* and *MDA5* were significantly downregulated by mutation of *KEAP1* or *STK11*. *STING* as an intracellular dsDNA sensor that activates the innate immune response (40), was observed among tumors with mutant *KEAP1* or *STK11* in recent studies (37, 39, 41). *STING* activation can lead to the production of type I interferons and other pro-inflammatory cytokines, which are crucial for the immune system’s recognition and elimination of cancer cells (42). *MDA5* is a crucial cytosolic RNA sensor that plays a pivotal role in the innate immune response by detecting viral infections and activating antiviral defenses (43, 44). Beyond its antiviral functions, emerging research underscores *MDA5*’s significance in cancer immunity, with its suppression being linked to immune evasion (45). Our study suggested that *MDA5* expression was also significantly suppressed by mutation in *KEAP1* or *STK11*. The mechanism by which *STING*/*MDA5* expression is altered by mutant *KEAP1* or *STK11* and their interaction with metabolic reprogramming remains unclear.

Redox homeostasis, as defined by the balance between reactive oxygen species (ROS) and antioxidants, has been intricately linked to the regulation of immune system. However, most of the previous studies centered on the direct impact of oxidative changes on biological functions of immune cells (46–51), but neglect their impact on the immunogenicity of tumor cells. Our study reveals a specific association between the that redox status and feature of immune evasion by specifically inhibiting *STING*/*MDA5*, characterized by the suppression of intrinsic STING/MDA5 expression and the interferon response specifically within tumor cells, a phenomenon not observed in other cell types of the TIME. Particularly, scRNA analysis showed that redox status of immune cells like macrophage, T cells etc. also correlated with reduced *STING*/*MDA5* expression in tumor cells and a suppressive TIME. These findings suggested that antioxidants derived from different cell types all contributed to the development of redox phenotype, which concurrently suppressed tumor immunogenicity by inhibiting *STING*/*MDA5* expression and interferon signaling of tumor cells. We recognize that the observed suppression of *STING/MDA5* due to redox alterations is primarily based on transcriptome data analysis, and further validation through *in vitro* experiments is required in the future.

The underlying mechanism by which redox phenotype suppressed *STING*/*MDA5* expression is yet to be explored in further studies. Previous studies have suggested a link between KEAP1-NRF2 pathway and *STING* suppression (52, 53). NRF2 is the master transcription factor that control the expression of a battery of genes involved in antioxidant response and detoxification processes (54). *KEAP1* negatively regulates NRF2 by directly binding and leading to its proteasomal degradation (55, 56). Loss-of -function mutations in *KEAP1* lead to constitute activation of NRF2 signaling (57). NRF2 has been reported as a negative regulator of *STING (*52, 53, 58), although the underlying mechanism remains a puzzle.

The most compelling clinical implication of our study lies in the potential for improved patient stratification and innovative trial design. The association between our redox signature and poor outcomes in the *KEAP1/STK11* double-mutant subgroup highlights a patient population in urgent need of better therapeutic options. These patients may be prioritized for more aggressive monitoring and considered for alternative treatment strategies beyond first-line immunotherapy. Looking forward, our findings advocate for the design of biomarker-driven clinical trials that specifically enroll patients with this high-risk molecular profile. Such trials could evaluate novel combinations, such as immunotherapy with (e.g., targeted redox-balancing agents or STING agonists), using our signature or the mutational status as an enrichment biomarker. This approach is essential to breaking the cycle of poor outcomes in this refractory population.

## Conclusions

In summary, our study elucidated the mechanism by which redox phenotype mediated immune evasion in NSCLC harboring *STK11* or *KEAP1* mutation. We first established a connection between redox phenotype and repression of pathways involved in dsDNA/dsRNA sensing, and clarify their association in suppressing immune infiltration. We findings also suggested that redox status predominantly suppressed *STING*/*MDA5* expression in tumor cells but not among other cell types within TIME. Our self-developed redox signature also may serve as a predictive biomarker for ICI responsiveness.

## Data Availability

The original contributions presented in the study are included in the article/**Supplementary Material**. Further inquiries can be directed to the corresponding authors.
