# Supplementary figures and images for: Redox phenotype confers T cell-exclusion microenvironment and resistance to immunotherapy by suppressing STING/MDA5 expression and interferon signaling in lung cancers harboring KEAP1/STK11 mutations
Source: Front Oncol. 2025 Nov 25;15:1676797. doi: 10.3389/fonc.2025.1676797 (PMC12685646; doi:10.3389/fonc.2025.1676797)

**sFig 1**

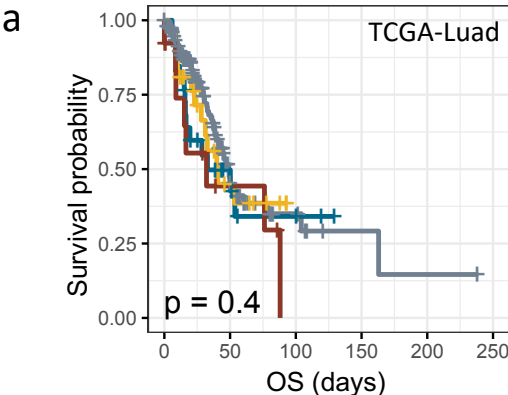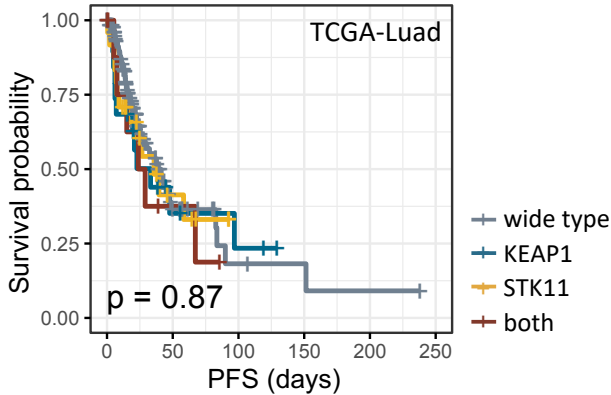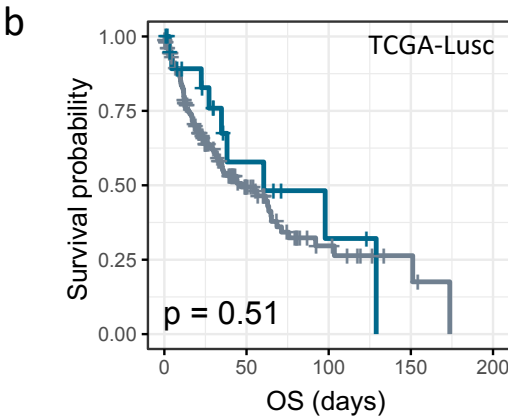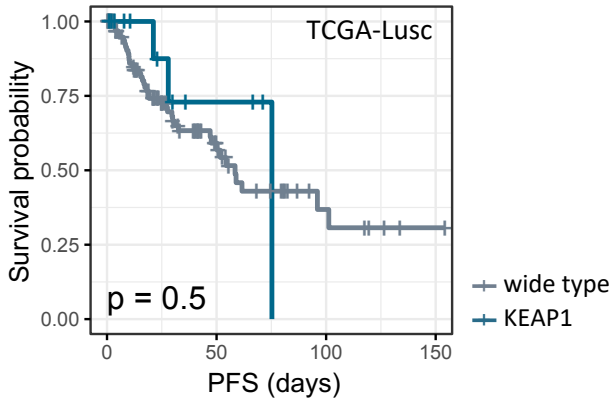

**sFig 2**

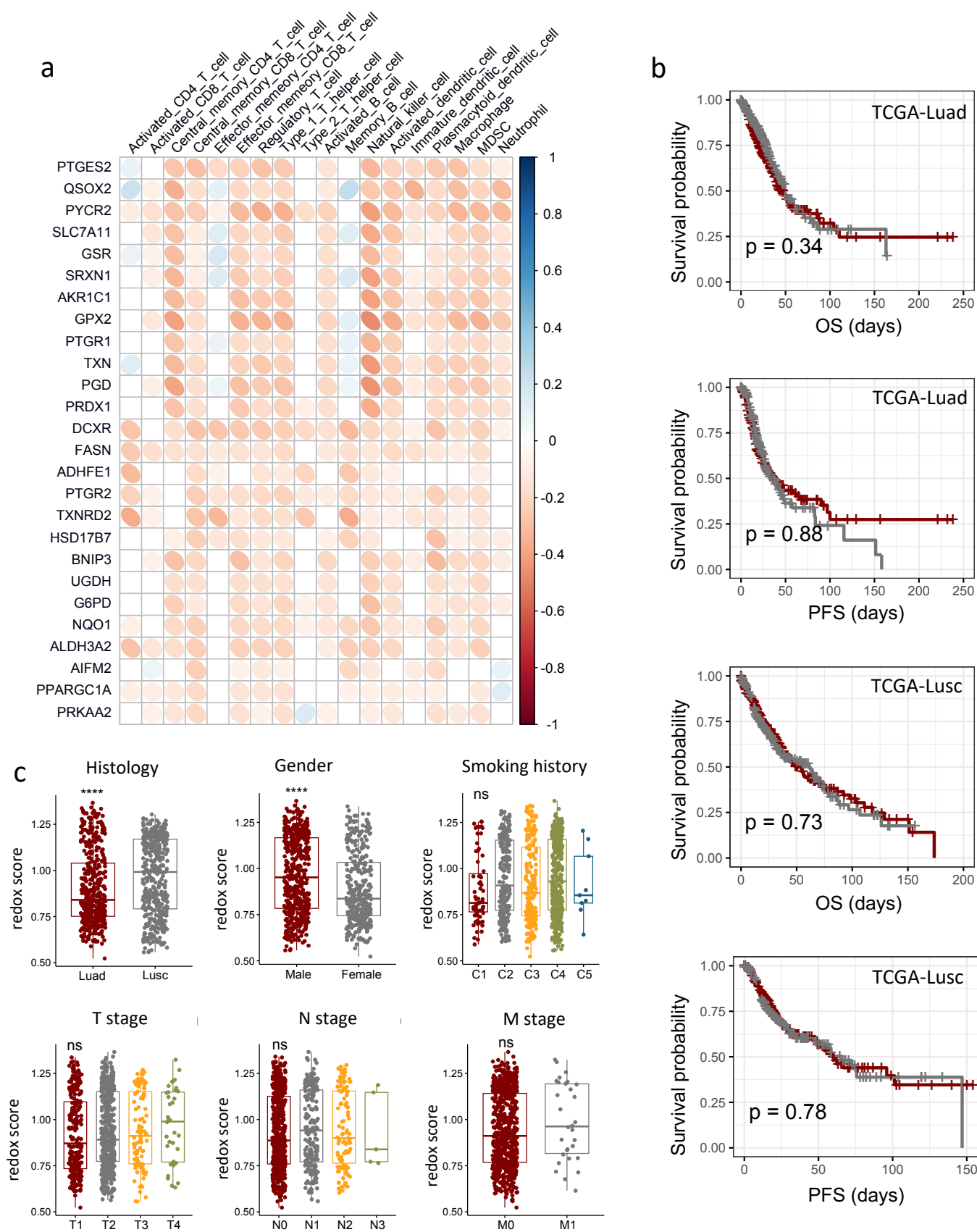

**sFig 3**

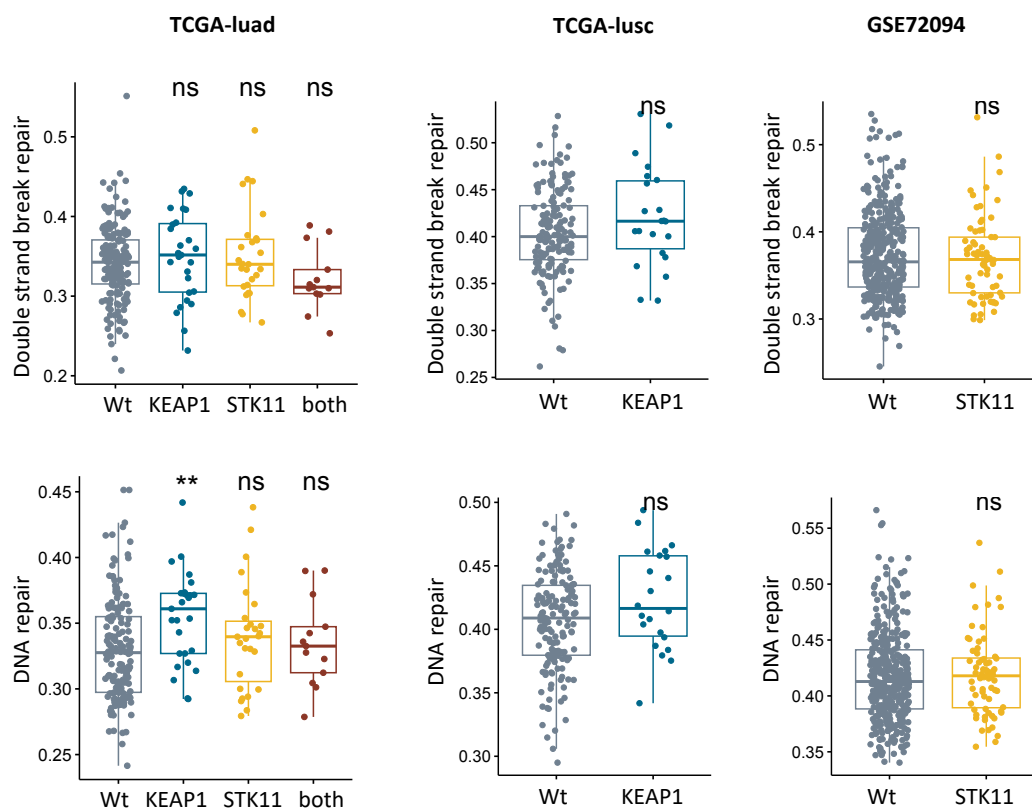

**sFig 4**

a

TCGA-luad

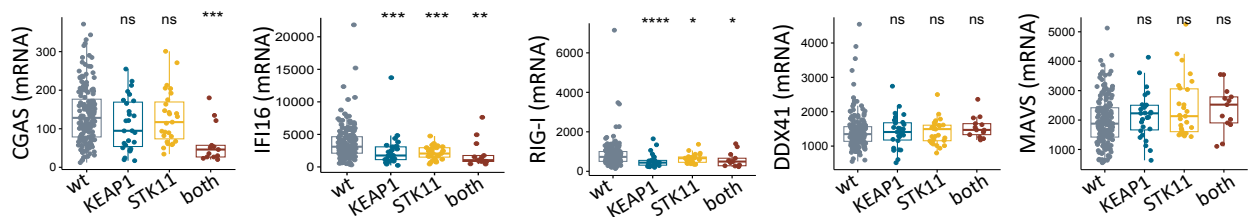

**b**

TCGA-lusc

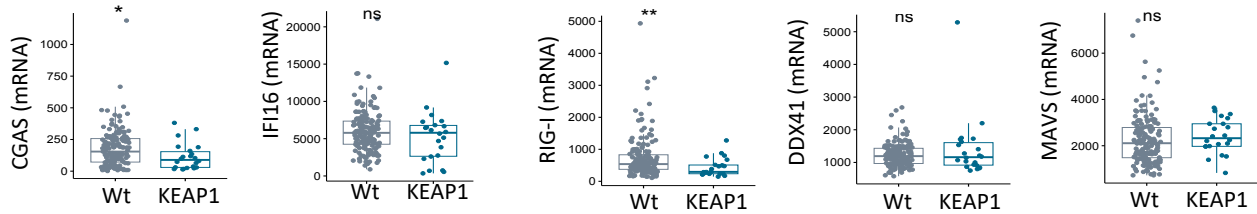

C

**GSE72094**

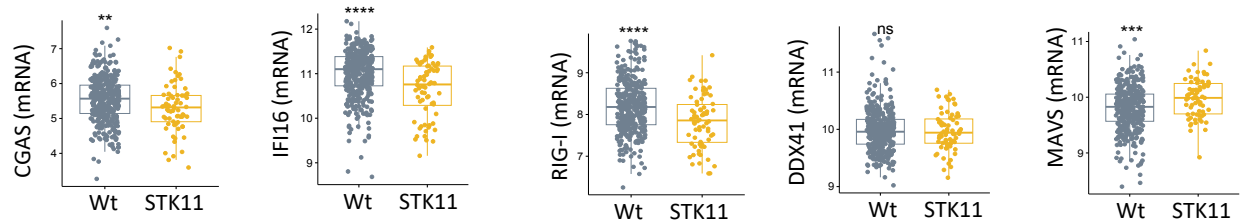

d

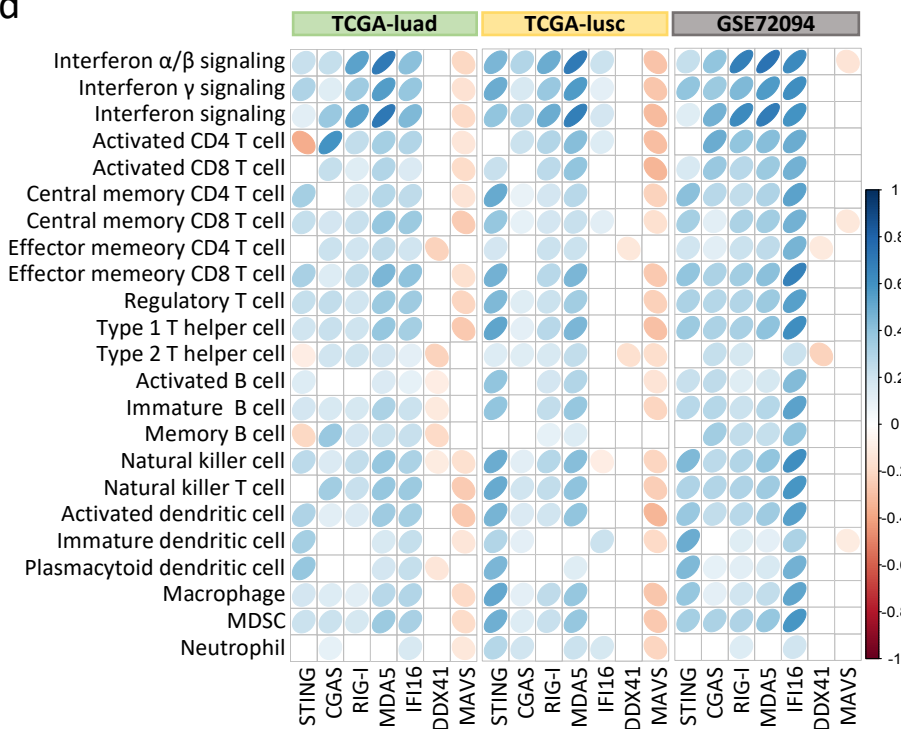

sFig 5

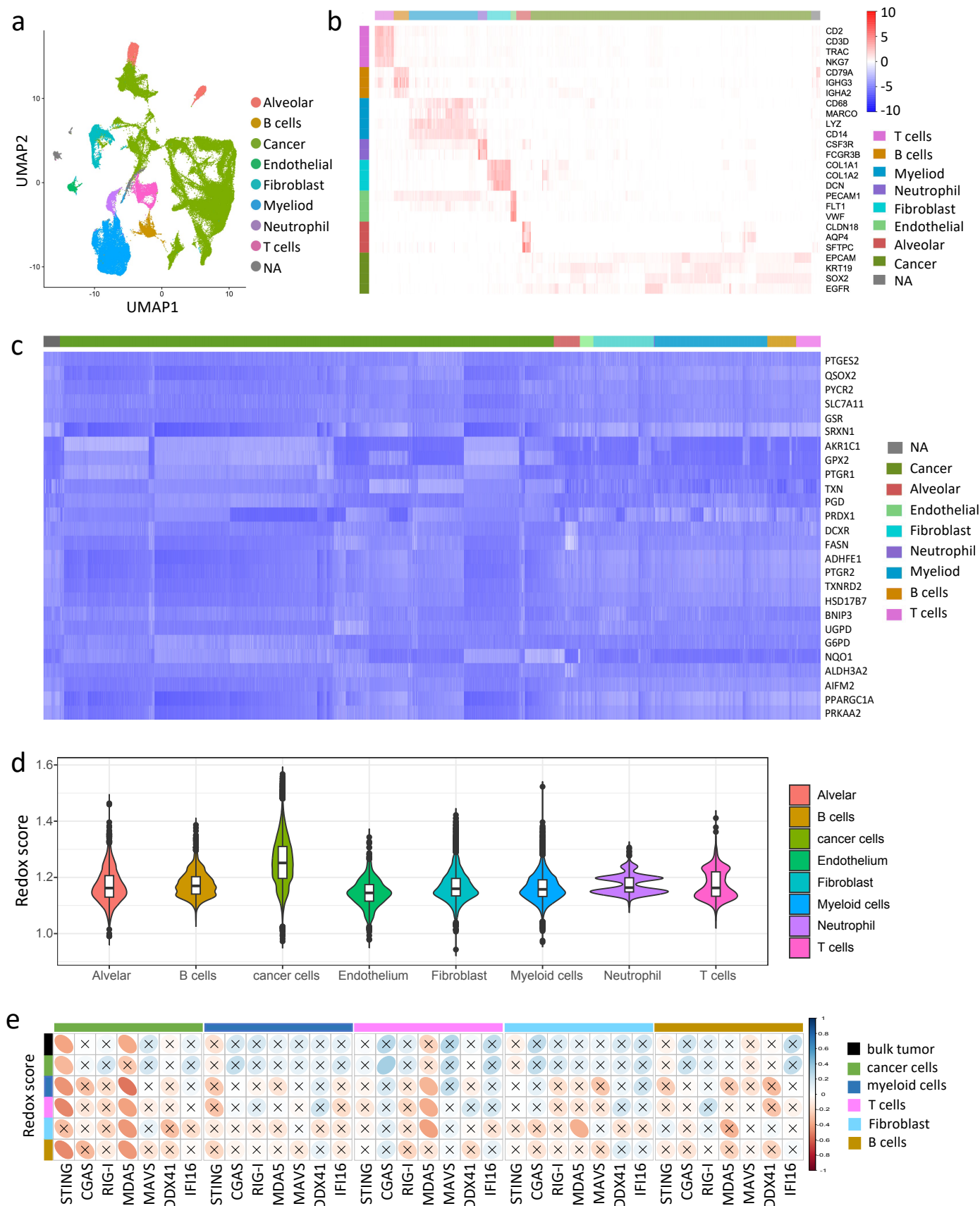

Supplement: Supplementary file 1 [file DataSheet1.pdf]
