# Supplementary material for: Redox phenotype confers T cell-exclusion microenvironment and resistance to immunotherapy by suppressing STING/MDA5 expression and interferon signaling in lung cancers harboring KEAP1/STK11 mutations
Source: Front Oncol. 2025 Nov 25;15:1676797. doi: 10.3389/fonc.2025.1676797 (PMC12685646; doi:10.3389/fonc.2025.1676797)
Supplement: Supplementary file 2 [file DataSheet2.docx]

# Supplementary figures and figure legends

# Figure legends of supplementary figures

## sFig 1. Prognostic significance of KEAP1/STK11 mutational status in NSCLC underwent surgical treatment

(a, b) Kaplan–Meier survival curves indicated mutational status of KEAP1 or STK11 didn’t impact OS or PFS among NSCLC patients received surgical treatment from TCGA-luad cohort (a) and TCGA-lusc cohort (b). As for TCGA-luad cohort, tumors were categorized into four groups based on mutational status of KEAP1 and STK11. As for TCGA-lusc cohort, tumors were grouped solely based on mutational status of KEAP1 as insufficient cases harboring mutant STK11.

## sFig 2. Correlation of redox signature with immune profiles, clinicopathogical features, and clinical outcomes of NSCLC underwent surgical treatment

a. Correlation matrix showing correlation between mRNA expression of genes derived from redox signature and infiltrating abundance of 20 immune cells in TCGA lung cancer cohort. Spearman Correlation analysis was performed, with blue ellipse obliquely upward representing positive correlation, and red ellipse obliquely downward representing negative correlation. The flatness of ellipse and the depth of the color represent the magnitude of the correlation (R value). Ellipse was presented only for those with correlation significance (P value) < 0.05.

b. Kaplan–Meier survival curves indicated no survival difference (OS and PFS) between with high redox group and low redox group among NSCLC patients received surgical treatment from TCGA-luad cohort and TCGA-lusc cohort. Tumors were categorized into high and low redox group with medium redox score as cut-off value.

c. Boxplots showing the difference in tumor redox score among patients with different histology, gender, smoking history, TNM staging (T staging, N staging and M staging) respectively. Wilcoxon tests was used to determined significance in difference between two groups. Kruskal -Wallis was performed for multiple comparison. *P < 0.05, **P < 0.01, ***P < 0.001.


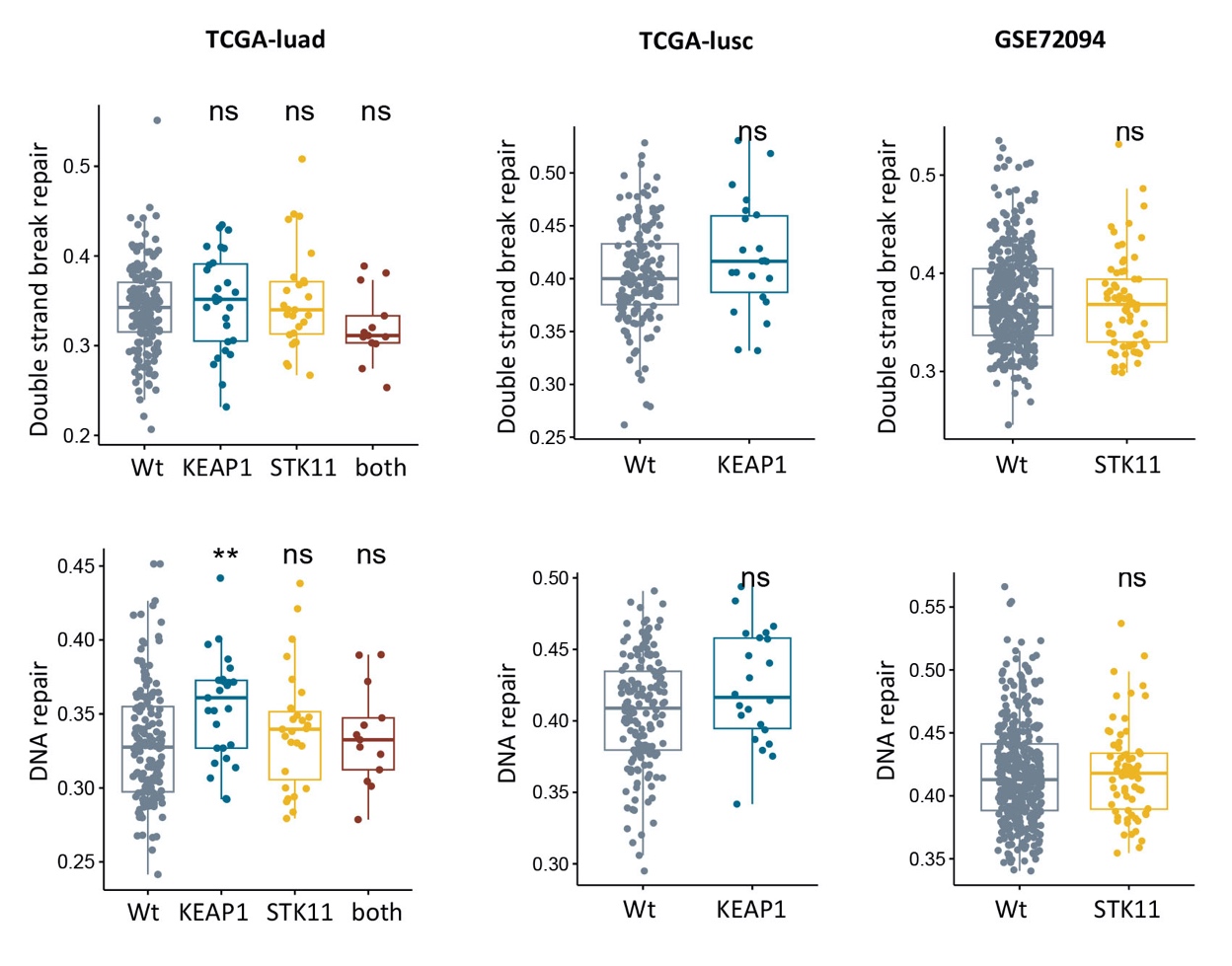


## sFig 3. Impact of KEAP1/STK11 mutations on DNA repairing pathways

Boxplots showing enrichment score of DNA repair pathway and Double strand break repair pathway between tumors with mutant or wide-type KEAP1/STK11 in TCGA-lusc cohort, TCGA-lusc and GSE72094 cohort. Data are presented as median with quartiles, and Wilcoxon tests was used to determined significance in difference between wild-type group and other groups. *P < 0.05, **P < 0.01, ***P < 0.001.

## sFig 4. Expressional status of genes involved in sdDNA/dsRNA/ssRNA sensing in NSCLC w/wt KEAP/STK11 mutations and their correlation with immune profiles

a-c. Boxplots showing expressional status of genes involved in dsDNA/dsRNA/ssRNA sensing among tumors with mutant or wide-type KEAP1/STK11 in TCGA-lusc cohort (a), TCGA-lusc (b) and GSE72094 cohort (c); Analysis only involved mutational status of KEAP1 or STK11 in TCGA-lusc cohort and GSE72094 cohort respectively, owning to insufficiency or unavailability of relevant data. Data are presented as median with quartiles, and Wilcoxon tests was used to determined significance in difference between wild-type group and other groups. *P < 0.05, **P < 0.01, ***P < 0.001.

d. Correlation matrix showing correlation between genes involved in dsDNA/dsRNA/ssRNA sensing and infiltrating abundance of 20 immune cells in three datasets (TCGA-luad, TCGA-lusc and GSE72094).

## sFig 5. scRNA analysis reveals expressional status of redox signature among different cellular clusters and their correlation with genes involved in sdDNA/dsRNA/ssRNA sensing

a. UMAP plot of 125,674 profiled cells from 42 samples labeled for different cell types.

b. Heatmap for visualization of the single-cell expression pattern of cell-type-specific gene markers.

c. Heatmap for visualization of single-cell expression pattern of 26 genes derived from the redox signature.

d. Violin plot showing redox score of different cell types.

e. Correlation matrix showing correlation between redox score of bulk tumor or different cellular cell type and mRNA expression level of genes involved in dsDNA/dsRNA/ssRNA sensing in different cell types. Spearman Correlation analysis was performed, with blue ellipse obliquely upward representing positive correlation, and red ellipse obliquely downward representing negative orrelation. The flatness of ellipse and the depth of the color represent the magnitude of the correlation (R value). The cross mark represents the failure of the significance test (P value > 0.05).
