# Supplementary material for: Redox phenotype confers T cell-exclusion microenvironment and resistance to immunotherapy by suppressing STING/MDA5 expression and interferon signaling in lung cancers harboring KEAP1/STK11 mutations
Source: Front Oncol. 2025 Nov 25;15:1676797. doi: 10.3389/fonc.2025.1676797 (PMC12685646; doi:10.3389/fonc.2025.1676797)
Supplement: Supplementary file 4 [file Table2.docx]

sTable 8.1. Cox Regression Analysis of PFS in Ravi lung cancer cohort

| variable | Univariate Analysis | |  | Multivariate Analysis | |
| --- | --- | --- | --- | --- | --- |
|  | HR [CI 5%-95%] | p.value |  | HR [CI 5%-95%] | p.value |
| Age | 0.98 [0.95-1.02] | 0.302 |  | 1.05 [0.96-1.14] | 0.284 |
| Gender | 0.63 [0.29-1.36] | 0.238 |  | 0.19 [0.03-1.37] | 0.0994 |
| Smoking history | 1.22 [0.6-2.46] | 0.584 |  | 18.39 [0.86-391.47] | 0.062 |
| Histology | 1.05 [0.43-2.61] | 0.911 |  | 0.07 [0.00-1.26] | 0.0708 |
| Stages | 0.94 [0.66-1.35] | 0.733 |  | 0.38 [0.08-1.85] | 0.2317 |
| PD-L1 | 0.92 [0.16-5.29] | 0.925 |  | 0.04 [0.00-2.06] | 0.1099 |
| KEAP1 | 1.14 [0.34-3.77] | 0.835 |  | 1.09 [0.01-78.87] | 0.9699 |
| STK11 | 0.65 [0.22-1.87] | 0.424 |  | 1.94 [0.01-366.50] | 0.804 |
| KRAS | 1.61 [0.74-3.48] | 0.229 |  | 30.64 [0.81-1163.71] | 0.0652 |
| TP53 | 1.78 [0.52-6.1] | 0.362 |  | 0.01 [0.00-2.34] | 0.1016 |
| Age | 0.98 [0.95-1.02] | 0.302 |  | 1.05 [0.96-1.14] | 0.284 |
| Redox level | 2.18 [1.03-4.62] | **0.0417** |  | 6.31 [0.63-63.24] | 0.1171 |

sTable 8.2. Cox Regression Analysis of OS in Ravi lung cancer cohort

| variable | Univariate Analysis | |  | Multivariate Analysis | |
| --- | --- | --- | --- | --- | --- |
|  | HR [CI 5%-95%] | p.value |  | HR [CI 5%-95%] | p.value |
| Age | 1.01 [0.96-1.05] | 0.78 |  | 1.05 [0.96-1.14] | 0.284 |
| Gender | 0.59 [0.22-1.62] | 0.307 |  | 0.19 [0.03-1.37] | 0.0994 |
| Smoking History | 1.64 [0.65-4.14] | 0.294 |  | 18.39 [0.86-391.47] | 0.062 |
| Histology | 0.62 [0.18-2.20] | 0.461 |  | 0.07 [0.00-1.26] | 0.0708 |
| Stages | 1.16 [0.66-2.06] | 0.609 |  | 0.38 [0.08-1.85] | 0.2317 |
| PD-L1 | 1.10 [0.04-31.41] | 0.956 |  | 0.04 [0.00-2.06] | 0.1099 |
| KEAP1 | 1.34 [0.17-10.25] | 0.779 |  | 1.09 [0.01-78.87] | 0.9699 |
| STK11 | 1.00 [0.23-4.42] | 0.998 |  | 1.94 [0.01-366.50] | 0.804 |
| KRAS | 0.47 [0.16-1.33] | 0.154 |  | 0.03 [0.00-1.24] | 0.0652 |
| TP53 | 0.00 [0.00-Inf] | 0.998 |  | 75.34 [0.43-13302] | 0.1016 |
| Redox level | 3.12 [1.09-8.94] | **0.0337** |  | 6.31 [0.63-63.24] | 0.1171 |

sTable 8.3. Logistic Regression Analysis of clinical response in Ravi lung cancer cohort

| variable | OR [CI 5%-95%] | p.value |
| --- | --- | --- |
| Age | 0.98 [0.90-1.05] | 0.57 |
| Gender | 1.14 [0.24-6.22] | 0.87 |
| Smoking History | 1.01 [0.98-1.06] | 0.44 |
| Histology | 2.69 [0.40-53.67] | 0.38 |
| Stages | 1.13 [0.48-2.35] | 0.75 |
| PD-L1 | 3.34 [0.05-138.96] | 0.52 |
| KEAP1 | 1.18 [0.06-9.64] | 0.89 |
| STK11 | 1.62 [0.07-14.97] | 0.69 |
| KRAS | 0.49 [0.07-2.46] | 0.42 |
| TP53 | 0.91 [0.04-6.97] | 0.94 |
| Redox level | 0.06 [0.00-15.35] | 0.33 |

sTable 9.1. Cox Regression Analysis of PFS in Gide melanoma cohort

| variable | Univariate Analysis | |  | Multivariate Analysis | |
| --- | --- | --- | --- | --- | --- |
|  | HR [CI 5%-95%] | p.value |  | HR [CI 5%-95%] | p.value |
| Age | 1.00 [0.97 -1.03] | 0.981 |  | 1.00 [0.98 -1.03] | 0.77048 |
| Gender | 0.59 [0.27 -1.31] | 0.198 |  | 1.23 [0.52 -2.93] | 0.63572 |
| PD-L1 | 0.19 [0.08 -0.43] | **<0.001** |  | 0.14 [0.06 -0.33] | **< 0.001** |
| Redox level | 2.34 [1.11 -4.97] | **0.0263** |  | 3.60 [1.54 -8.41] | **0.00306** |

sTable 9.2. Cox Regression Analysis of OS in Gide melanoma cohort

| variable | Univariate Analysis | |  | Multivariate Analysis | |
| --- | --- | --- | --- | --- | --- |
|  | HR [CI 5%-95%] | p.value |  | HR [CI 5%-95%] | p.value |
| Age | 0.99 [0.96-1.02] | 0.459 |  | 0.99 [0.96-1.02] | 0.5081 |
| Gender | 0.32 [0.11-0.93] | 0.0369 |  | 0.79 [0.24-2.58] | 0.6902 |
| PD-L1 | 0.17 [0.07-0.46] | **<0.001** |  | 0.16 [0.06-0.46] | **<0.001** |
| Redox level | 2.40 [1.03-5.58] | **0.0426** |  | 2.68 [1.08-6.63] | **0.0328** |

sTable 9.3. Logistic Regression Analysis of clinical response in Gide melanoma cohort

|  | Univariate Analysis | |  | Multivariate Analysis | |
| --- | --- | --- | --- | --- | --- |
| Variable | OR [CI 5%-95%] | p value |  | OR [CI 5%-95%] | p value |
| Age | 1.00 [0.96-1.06] | 0.723 |  | 1.02 [0.96-1.08] | 0.531 |
| Gender | 2.4 [0.67-9.23] | 0.187 |  | 1.03 [0.17-5.47] | 0.976 |
| PD_L1 | 10 [2.54-47.89] | 0.0018 |  | 15.79 [3.10-130.96] | 0.0027 |
| Redox level | 0.26 [0.07-0.93] | 0.044 |  | 0.16 [0.02-0.80] | 0.040 |

Notes: HR, hazard ratios; OR, odds ratios; CI, confidence interval
